# Efficacy of a combined contraceptive regimen consisting of condoms and emergency contraception pills

**DOI:** 10.1186/1471-2458-14-354

**Published:** 2014-04-14

**Authors:** Rui Zhao, Jun-Qing Wu, Yu-Yan Li, Ying Zhou, Hong-Lei Ji, Yi-Ran Li

**Affiliations:** 1 Shanghai Institute of Planned Parenthood Research/WHO Collaborating Center on Human Research, Shanghai 200032, China; 2 National Laboratory of Contraceptives and Devices Research of China, Shanghai 200032, China

**Keywords:** Contraception, Methods, Pregnancy rates, Cumulative life table rates, Women

## Abstract

**Background:**

To evaluate and compare the effectiveness of the combined regimen (consisting of condoms and emergency contraception pills (ECP)) and using condoms only for the purpose of preventing pregnancy.

**Methods:**

One-thousand-five-hundred-and-sixty-two (1,562) couples as volunteers enrolled at nine centers in Shanghai. Eight-hundred-and-twelve (812) were randomized to use male condoms and ECP (i.e., Levonorgestrel) as a back-up to condoms (the intervention group) and 750 to use male condoms only(the control group), according to their working unit. Participants were visited at admission and at the end of 1, 3, 6, 9, and 12 months. The cumulative life table rates were calculated for pregnancy and other reasons for discontinuation.

**Result:**

The gross cumulative life table rates showed that the cumulative discontinuation rates for all reasons during the year of follow-up in the condoms plus emergency contraception group and the condoms only group were 7.76 ± 0.94 and 6.61 ± 0.91, respectively, per 100 women (χ2 = 0.41, p = 0.5227). The cumulative gross pregnancy rate of the condoms plus emergency contraception group and the condoms only group were 2.17 ± 0.52 and 1.25 ± 0.41, respectively, per 100 women (χ2 = 1.93, p = 0.1645). The Pearl Index in the condoms plus emergency contraception group and the condoms only group were 2.21% and 1.26%, respectively.

**Conclusion:**

Male condoms remain a highly effective contraceptive method for a period of one year while consistently and correctly used. In addition, the lowest pregnancy rate followed from perfect use condom.

## Background

A large number of studies have found that the consistent and correct use of condoms is by far the most important factor in preventing both pregnancy [1-8] and sexually transmitted disease (STD) [9,10], including HIV [11]. However, a limited but growing body of research literature indicated that most of these studies were conducted from a disease-prevention perspective, neglecting the fact that condoms were originally created as a method to prevent unwanted pregnancy [12].

To our knowledge, few relevant randomized and controlled clinical trials were conducted to test the efficacy of latex condom in China. In Shanghai, more than 20% of married women at reproductive age relied on male latex condoms to protect against unintended pregnancy and STDs in 2008 [13]. And recently, more and more women at reproductive age choose latex condoms as a contraception method. This rate rose to 31.04% in 2012 [14]. Since this is the major contraceptive method for married women in Shanghai, the contraceptive efficacy of latex condom needs to be further tested.

Considering ethical issues, it is hard to carry out clinical trials in which volunteers do not use condoms. Therefore, we created a contrast between the control group with male condoms only and the intervention group with a combined contraceptive method consisting of condoms and emergency contraceptive pills (ECPs). ECPs are certainly not 100% effective, but when taken within 72 hours of unprotected sex, timely emergency contraception may reduce women’s risk of unintended pregnancy by 89–95% [15]. Moreover, there is no known harm to the woman, the course of her pregnancy, or the fetus if ECPs are accidentally used [16,17].

Therefore, a follow-up study to this study was designed to evaluate and contrast the effectiveness of the combined regimen with the method using condoms only in preventing pregnancy.

## Methods

### Subjects and procedure

The study began in October 2003 and was completed in December 2007. One-thousand-five-hundred-and-sixty-two (1,562) couple-volunteers were enrolled at nine centers in Shanghai. Eight-hundred-and-twelve (812) were enrolled to the intervention group (use male condoms and ECP (i.e., Levonorgestrel) as a back-up to condoms) and seven-hundred-and-fifty (750) with the control group (male condoms only), according to their working unit. Women were eligible for this study if younger than 35 years (exclusive), in good health, with regular menstrual periods, sexually active in an ongoing relationship, at risk for pregnancy, with the last menstrual period on the expected date, willing to use the combined regimen/condoms as contraception for 13 months, willing to keep a daily diary recording menstrual pattern and detailed information regarding each sexual intercourse (SI) encounter, specifically using condoms/ECP in acts of SI, and willing to return the daily diary to the clinic on a monthly basis and to return to the clinic for a short interview at 1, 3, 6, 9, and 12 months following enrollment. All participants were informed of the study’s content.

The baseline information of the two groups was collected under permission. The routine follow-up visits were scheduled at the ends of the 1^st^, 3^rd^, 6^th^, 9^th^, and 12^th^ months after admission. Subjects were instructed to return to the clinic at any time for any problem that might be related to the use of the study condoms, the ECP, or participation in the study in general. At each scheduled visit, diary cards were reviewed for completeness of information on menses, acts of intercourse (subjects should accurately record the time of every situation of SI), condom use and, if applicable, ECP use. During the follow-up visits, subjects were interviewed using a brief questionnaire. A sufficient supply of condoms and emergency contraception was provided to the participants in the intervention group, and subjects in the control group were provided sufficient condoms. Both condoms and ECP were free of charge during the study period.

### Study materials

The condoms used in the study were World Health Organization (WHO)-approved standard thin, yellow, silicone lubricated latex condoms. The condoms were produced by SEDHUNG IND. CO., LTD., KOREA. The width and single-wall thickness were 53.0 ± 1.0 mm and 0.07 ± 0.01 mm, respectively. In addition to condoms, couples allocated to the condoms plus emergency contraception group used an ECP (i.e., Levonorgestrel) as a back-up to condoms. Women allocated to the condom/ECP combined regimen group were provided with Levonorgestrel to use in the event of breakage, leakage, slippage, or other failures, which was white pill and produced by Germany. The dose of 0.75 mg Levonorgestrel in the study was taken within 48–72 hours of unprotected coitus, and the same dose was repeated 12 hours later. Training and education material providing information on the correct use of condoms and the emergency contraception regimen was prepared and used by the participating clinics during the course of the study.

### Discontinuation from the study

Subjects could discontinue participation in the study at any time for any personal or medical reason, such as moving away from the study area, asked by husbands, breakage of condom, menstrual problems, plan for pregnancy, side effects, or loss for follow-up. The reasons for discontinuation and side effects were recorded.

### Study outcome measures

Pregnancy was the primary outcome index; it was diagnosed using highly sensitive urine tests and was confirmed by physical examination and the use of a B-ultrasonic machine in the hospital. Other outcomes, such as condom breakage, slippage, bleeding, and so on were recorded by the subjects. The occurrence of adverse experiences and reasons for subjects’ discontinuation were monitored throughout the study.

### Statistical analysis

Data on all the records were entered twice by different professionals using EpiData 3.1 to enable a comparison between the data and the correction of data entry mistakes. Data cleaning included consistency verification for all variables. Data analyses were carried out via the SAS 9.1.3 package (SAS Institute). Analyses were conducted for each outcome of interest. Analyses focused on the differences between two groups. Descriptive statistics included mean, frequencies, and proportions. Chi-square analysis was used to analyze the distribution of subjects’ characteristics. The log-rank test was used to analyze the cumulative condom use rate and cumulative termination rate between two groups. The gross cumulative life table rates were calculated for pregnancy and other reasons of discontinuation. In addition, in this study, P < 0.05 was considered statistically significant.

## Results

### Baseline characteristics

Of the 824 women enrolled, 812 were eligible for admission into the intervention group, and 749 subjects were interviewed five times. Of the 754 women enrolled in the control group, 750 were eligible and 699 were interviewed five times. The study was conducted in nine urban districts of Shanghai. Of the 812 subjects in the intervention group, there were 749 subjects who finished the whole study. The follow-up rate was 92.24% in the intervention group. Of the 750 subjects in the control group, there were 699 subjects who finished the study. The follow-up rate in the control group was 93.20%. There were no statistically significant differences between follow-up rates of two groups (p > 0.05).

The mean ages of the subjects in both groups were near 30 years old. The mean marriage length was 5.4 years in the intervention group and 5.5 years in the control group. The mean age of subjects and husbands, education distribution of subjects and husbands, and husbands’ occupation distribution in the intervention group and control group were not significantly different. As showed in Table 1, there was a majority (58.3% and 61.3%, respectively) of subjects in the intervention group and the control group with a high school education. Most of the subjects’ husbands (49.4% of the intervention group and 53.0% of the control group) had a high school educational level. The distribution of the subjects’ occupations between the two groups was slightly different. 41.5% of the subjects were workers in both groups. 39.2% and 42.0% of husbands in the intervention group and the control group, respectively, were workers.

**Table 1:** Distribution of subjects’ characteristics by group (%)

**Variables**	**Intervention group (n = 812)**	**Control group (n = 750)**	χ^**2**^	**P-value**
**Education level**				
**≤Primary**	0.1	0.8	8.89	0.064
**Secondary**	16.4	15.2		
**High school**	58.3	61.3		
≥**College**	14.7	15.2		
≥**University**	10.5	7.5		
**Husband’s education**				
**≤Primary**	0.3	0.7	4.73	0.317
**Secondary**	12.0	11.7		
**High school**	49.4	53.3		
**College**	20.8	18.1		
≥**University**	17.5	16.2		
**Occupation**				
**Doctor**	11.2	7.3	14.36	0.006
TSS*	11.8	15.3		
**Official**	13.3	10.4		
**Worker in factory**	41.5	41.5		
Others**	22.2	25.5		
**Husband’s occupation**				
**Doctor**	2.6	2.3	2.42	0.660
**TSS**	17.1	15.6		
**Official**	17.7	15.9		
**Worker in factory**	39.2	42.0		
Others**	23.4	24.2		
**Who live together with**				
**Children**	53.3	55.2	2.05	0.916
**Children + parent**	18.5	17.6		
**Husband only**	19.3	17.7		
**Others**	8.9	9.5		
**Bedroom**				
**With husband**	62.1	52.5	17.11	0.001
**With children**	36.7	44.7		
**Others**	1.2	2.8		

*TSS: Teacher or scientific staff; ** Others: self-employed staff, service workers and shop employees.

As for housing conditions, the distribution of family member number in the intervention group and the control group was not significantly different. Regarding bedroom conditions, 62.1% of the subjects in the intervention group and 52.5% of those in the control group had their own living room. There were 36.7% and 44.7% subjects in the intervention group and the control group, respectively, living with their children in the same bedroom; there were 1.2% and 2.8% subjects in the intervention group and the control group, respectively, sharing a living room with others. The distribution difference of bedroom conditions between the two groups was statistically significant (p = 0.001).

Before the study, there were 92% and 88.5% of the subjects in the intervention group and the control group, respectively, who didn’t know how to use condoms correctly. After they were enrolled into the study, the staff provided guidance on condom use and made sure that every subject could use condoms correctly.

### Discontinuation

Among all discontinuations, only 4.76% (3/63) of the subjects in the intervention group and 5.88% (3/51) of the subjects in the control group who withdrew from the study because of reasons related to the condoms (allergic reaction and condom breakage), while a large part, 38.1% (n = 24/63) of the intervention group and 31.4% (n = 16/51) of the subjects in the control group, withdrew voluntarily from the study because of personal reasons. In addition, 27.0% (n = 17/63) and 17.6% (n = 9/51) of the discontinuations in the intervention group and the control group, respectively, were attributed to subjects’ pregnancies. Furthermore, among all pregnancies, 19.23% (n = 5/26) were because of condom breakage, 11.11% (n = 3/26) were due to forgetting to use a condom, 50% (13/26) were because of incorrect condom use, and 19.23% (n = 5/26) were due to the spouses’ dislike of condom use and other reasons. No subjects withdrew from the study because of condom slippage or bleeding.

Table 2 shows the cumulative condom use rate and cumulative termination rate in both groups. The gross cumulative life table rates showed that the cumulative discontinuation rates for all reasons during the year of follow-up in the intervention group and the control group were 7.76 ± 0.94 and 6.61 ± 0.91, respectively, per 100 women. The log-rank test showed that there were no statistically significant differences between the discontinuation rates of the two groups (χ2 = 0.41, p = 0.5227).

**Table 2:** The condom cumulative use rate

**Intervention group**							**Control group**					
**Month**	**No. Subject at beginning**	**No. Sub not use condom**	**Terminate**	**Cumulative condom use rate**	**Cumulative terminate rate**	**SE of Cumulative condom use rate**	**No. Subject at beginning**	**No. Sub not use condom**	**Terminate**	**Cumulative condom use rate**	**Cumulative terminate rate**	**SE of Cumulative condom use rate**
**X**	**N**_**x**_	**Wx**	**Tx**	***P***_***(x+1)***_	**Q**_**(x+1)**_	**Sp**_**(x+1)**_	**N**_**x**_	**Wx**	**Tx**	***P***_***(x+1)***_	**Q**_**(x+1)**_	**Sp**_**(x+1)**_
**0**	812	0	12	0.98522	0.01478	0.004238	750	0	11	0.98533	0.01467	0.004384
**1**	800	0	7	0.97660	0.02340	0.005304	739	1	6	0.97733	0.02267	0.005433
**2**	793	0	14	0.95936	0.04064	0.006931	732	0	5	0.97065	0.02935	0.006162
**3**	779	0	7	0.95074	0.04926	0.007594	727	0	5	0.96398	0.03602	0.006805
**4**	772	0	4	0.94582	0.05418	0.007944	722	0	3	0.95997	0.04003	0.007159
**5**	768	0	5	0.93966	0.06034	0.008356	719	0	4	0.95463	0.04537	0.007601
**6**	763	0	2	0.93719	0.06281	0.008514	715	0	4	0.94929	0.05071	0.008014
**7**	761	0	4	0.93226	0.06774	0.008818	711	0	2	0.94662	0.05338	0.008211
**8**	757	0	3	0.92857	0.07143	0.009037	709	0	4	0.94129	0.05871	0.008587
**9**	754	0	3	0.92488	0.07512	0.009250	705	0	0	0.94129	0.05871	0.008587
**10**	751	0	1	0.92365	0.07635	0.009319	705	0	1	0.93995	0.06005	0.008678
**11**	750	0	0	0.92365	0.07635	0.009319	704	0	2	0.93728	0.06272	0.008857
**12**	750	0	1	0.92242	0.07758	0.009388	702	0	3	0.93328	0.06672	0.009116
**Total**	**—**	**0**	**63**	**— —**	**— —**	**— —**	**— —**	**1**	**50**	**— —**	**— —**	**— —**

### Frequency of intercourse and condoms use

The frequency of intercourse between the intervention group and the condom only group was 60.7 ± 17.9 and 58.5 ± 18.7, respectively, per year. There was statistical significance between the two groups, and the frequency of the intervention group was higher than the condom only group (t = 2.3, p = 0.023). The frequency of condom use throughout intercourse between the intervention group and the condom only group was 58.1 ± 18.5 and 56.4 ± 19.7, respectively, per year. There was no statistical significance between the two groups (Table 3).

**Table 3:** Frequency of intercourse and condoms use per year

**Variable**	**Intervention group**	**Control group**	**t**	**P-value**
	**N ± SD**	**N ± SD**		
Intercourse frequency (n)	60.7 ± 17.9	58.5 ± 18.7	2.27	0.023
Frequency of condom use (n)	59.5 ± 17.9	57.4 ± 19.0	2.14	0.033
Frequency of condom use throughout intercourse (n)	58.1 ± 18.5	56.4 ± 19.7	1.69	0.091
Frequency of check on condom after intercourse (n)	57.6 ± 19.2	55.9 ± 19.4	1.66	0.099

### Pregnancy rate

The gross cumulative life table rates were calculated for pregnancy and other reasons for discontinuation. Table 4 shows the gross pregnancy rates of the two groups. The cumulative gross pregnancy rates in the intervention group and the control group were 2.17 ± 0.52 and 1.25 ± 0.41, respectively, per 100 women. The log-rank test for the events of pregnancy in the intervention group and the control group showed that there were no statistically significant differences in follow-up between the two groups (χ2 = 1.93, p = 0.1645).

**Table 4:** The gross rate of pregnancy between the two groups

**Intervention group**						**Control group**				
**Month**	**No. Sub. Of not use condom**	**No. Preg.**	**Cumulative condom use rate**	**Cumulative of pregnancy gross rate**	**SE of Cumulative pregnancy gross rate**	**No. Sub. Of not use condom**	**No. Preg.**	**Cumulative condom use rate**	**Cumulative of pregnancy gross rate**	**SE of Cumulative pregnancy gross rate**
**X**	***W*x**	***E*x**	**P**_**(x)**_	**Q**_**e(x+1)**_	**S**_**qe(x+1)**_	***W*x**	***E*x**	**P**_**(x)**_	**Q**_**e(x+1)**_	**S**_**qe(x+1)**_
0	0	4	0.99505	0.00495	0.002469	0	0	1.00000	0.00000	0.000000
1	0	1	0.99381	0.00619	0.002764	1	1	0.99864	0.00136	0.001358
2	0	2	0.99128	0.00872	0.003283	0	1	0.99727	0.00273	0.001926
3	0	1	0.99000	0.01000	0.003519	0	0	0.99727	0.00273	0.001926
4	0	1	0.98872	0.01128	0.003742	0	2	0.99451	0.00549	0.002738
5	0	2	0.98614	0.01386	0.004153	0	1	0.99313	0.00687	0.003065
6	0	1	0.98484	0.01516	0.004344	0	0	0.99313	0.00687	0.003065
7	0	1	0.98354	0.01646	0.004528	0	1	0.99173	0.00827	0.003365
8	0	0	0.98354	0.01646	0.004528	0	1	0.99033	0.00967	0.003640
9	0	3	0.97963	0.02037	0.005042	0	0	0.99033	0.00967	0.003640
10	0	1	0.97833	0.02167	0.005201	0	0	0.99033	0.00967	0.003640
11	0	0	0.97833	0.02167	0.005201	0	2	0.98752	0.01248	0.004138
12	0	0	0.97833	0.02167	0.005201	0	0	0.98752	0.01248	0.004138
**Total**	**0**	**17**	— —	— —	— —	**1**	**9**	— —	— —	— —

### Pearl Index between the two groups

The Pearl index is the ratio of the number of pregnancies observed from the study divided by the total number of person year of all study participants observed. The total person months in the intervention group were 9268.5 and 8639.5 in the control group. The Pearl Index in the intervention group was 2.21% (17/9268.5*12) and 1.26% (9/8639.5*12) in the control group (Table 5).

**Table 5:** Pearl Index at different follow-up periods

**Month**	**Intervention group**			**Control group**			**Total**		
	**Person-month**	**No. of preg.**	**Pearl Index**	**Person-month**	**No. of preg.**	**Pearl Index**	**Person-month**	**No. of preg.**	**Pearl Index**
0 ~ 2	2383.2	7	3.52	2479.7	2	0.97	4862.9	9	2.22
3 ~ 5	2307.6	4	2.08	2060.8	3	1.75	4368.4	7	1.92
6 ~ 8	2275.2	2	1.05	2036.9	2	1.18	4312.1	4	1.11
9 ~ 11	2256.8	4	2.13	2019.5	2	1.19	4276.3	6	1.12
0 ~ 11	9222.8	17	2.21	8596.9	9	1.26	17819.7	26	1.75

### Multiple Cox regression

All factors (including the subjects and their husbands’ ages, occupations, menstruation status, intercourse frequency, contraception regimen and use of condoms experience) that may impact the pregnancy occurring were included in the multiple Cox regression analysis. After controlling for potential confounding variables, the multiple Cox regression of pregnancy showed that discontinuation because of pregnancy was associated with the variables of husband age, the subjects’ occupations, and the number of contraceptive methods used before the study. Women whose husbands were 30 ~ 34 and ≥35 years old had a lower risk of pregnancy (RR = 0.812 and RR = 0.765) compared with those subjects whose husbands were under 30 years old. Compared with subjects who were medical staff, workers, self-employed staff, and shop employees might have higher risk of pregnancy, while teachers and technician and officials had no significant difference of risk in the model. The more contraceptive methods used by subjects before the study, the higher risk of pregnancy the subjects might have (Table 6).

**Table 6:** Multiple Cox regression of discontinuation occurring because of pregnancy

**Variable**	**Reference Group**	**Comparative Group**	**β**	**SE**	**Wald** χ**2**	**p**	**RR**	**RR(95%CI)**
**Husband age**	**<30**	30 ~ 34	-0.2078	0.0608	10.19	0.0014	0.812	0.921 ~ 0.915
		≥35	-0.2681	0.0719	13.90	0.0002	0.765	0.409 ~ 0.881
**Occupation**	Medical staff	TST*	0.0987	0.1093	0.82	0.3665	1.100	0.891 ~ 1.367
		Official	0.1257	0.1124	1.25	0.2633	1.130	0.910 ~ 1.413
		Factory worker	0.2350	0.0942	6.23	0.0125	1.265	1.052 ~ 1.521
		Others**	0.2115	0.0994	4.53	0.0333	1.236	1.017 ~ 1.051
**No. of contra. used**			0.1569	0.0435	13.01	0.0003	1.170	1.074 ~ 1.274

*TST-Teacher or scientific staff; **others: individual owner, service workers and shop employees.

## Discussion

Male condoms in various forms have been used for centuries [18]. Latex male condoms have been mass-produced since the mid-1800s [19], and since the 1930s, latex condoms have been available to prevent both pregnancy and sexually transmitted disease [20]. Up till now, condoms are one of most popular contraceptive methods and are widely used in many nations for contraception. This randomized and controlled study had several strengths in the efficacy evaluation between two groups. It provides considerable evidence that male condom use remains a highly effective contraceptive method for a period of one year if consistently and correctly used. In this study, over the course of the whole year, the cumulative gross pregnancy rates in the intervention group and the condom only group were, respectively, 2.17% and 1.25%. The Pearl Index intervention group and condom only group were, respectively, 2.21% and 1.26%.The results of our study indicated a low pregnancy rate (2.17% and 1.25%, respectively). However, when we reviewed much of the literature, we found that the reported pregnancy rates for condom users involve a broader range. Some studies showed that the clinical failure rate of male latex condoms rarely exceeded 2% [21-23]. But other studies found that the pregnancy risk with condoms is high (18.4% in Thailand, 29.5% in Uganda, and 23.3% in Zimbabwe) [3]. After reviewing and discussing the relevant literature, we found the lower pregnancy rates (2.17% and 1.25%, respectively) of our study are primarily due to the study design. Those studies with high pregnancy rates were always inferred from cross-sectional survey studies. But in the randomized and controlled studies in which latex condoms were usually used as the control methods compared with other methods, the pregnancy rate is at a low level, just like in our study. In a relevant study, the pregnancy rate of the six-cycle consistent-use of latex condoms was 1.0% [5]. Another completed randomized controlled trial of condom use reported that the pregnancy rate was 1.2 per 100 women using latex condoms in six-months of consistent use (subjects used condoms in every instance of sexual intercourse) [24]. The similarity between those trials and our study represents a ‘correct’ use of condoms, including consistent and correct condom use.

Some studies [25,26] indicated that the participants should be educated in correct condom use, but others indicated that education is not related with correct condom use and that the benefits of written instructions are limited and do not fully teach correct condom use [27]. Our study suggested that providing one-on-one training and counseling to subjects about condom use might be crucial to encouraging the consistent and correct use of condoms. Before the study, we asked the subjects: “Do you know how to use condoms correctly?” If the answer was “no,” we explained and gave detailed training on the usage of condoms after interviewing. If the answer was “yes” or “not sure,” we let the subject describe how to use a condom and checked points according to the subject’s description by drawing a “√” at the mentioned point (the point included: a. a condom should be used for every instance of sexual intercourse; b. check if condom is broken before using it; c. exhaust the air from the top bubble; d. hold the base of the condom while drawing the penis out of the vagina; e. if breakage, leakage, or slippage occurs, an emergency method should be used). But the results make us regret that there were 92% and 88.5%, respectively, among our subjects who didn’t know how to use condoms completely correctly. Other studies also obtained a similar result. In a study conducted in Africa [28], taking all criteria together, only 11% of participants performed a correct condom use demonstration. It is obvious that subjects need more information and guidance about how to correctly and consistently use condoms and how to manage breakage, slippage and other relative problems. During the project, therefore, we conducted much training to teach subjects (the couples together) correct condom use and also emphasized the importance of consistent use. After the training, we conducted a small test to test whether they could answer the questions right or not, meanwhile, we provided condoms and penis moulds to subjects so they could demonstrate the process of condom use until they could do it correctly. Finally, we found that the one-on-one training and counseling was truly helpful to the subjects. The lower pregnancy rates (2.17% and 1.25%, respectively) of our study may support the idea.

At the beginning of the study, we assumed that the combined contraceptive regimen consisting of condoms and emergency contraception pills would be helpful to improve the contraceptive effect. However, the log-rank test results show that there wasn’t significant difference between the intervention group and the condom only group in preventing pregnancy in our study, and the Cox regression also illustrated that the contraception regimen show no effect on pregnancy rate. This may result from the situation that pregnancies reported with condom use are almost always due to inconsistent and incorrect use and not to defective condoms [29] and that failure of the condom itself is rare (the reported breakage or slippage rate of condoms is 7.9%) [30]. In our study, there were 4.76% and 5.88% respectively subjects who discontinued participation in the study because of condom reasons, such as condom breakage or discomfort. Among the 26 pregnancies (17 in the intervention group and nine in the control group), only five pregnancies were due to condom-related reasons. As has been shown from this and the other relevant studies mentioned above, once a person starts using condoms regularly, correctly, and consistently, condoms may play a more significant role in pregnancy prevention and in reducing the pregnancy rate to a very low level. So it is not strange that a significant difference between the two groups was not found in this study.

In this study, we carried out a follow-up study to collect detailed information on the subjects and adopted cumulative life table rates to calculate pregnancy occurrence and other variables. The result of this study is expected to provide useful information on the contraceptive effectiveness of condoms and the combined regimen consisting of condoms and ECPs. In this population, it seemed that the combined contraceptive regimen did not improve contraceptive effectiveness. However, this study also found that the rates of non-use of condoms and incorrect use of condoms among our subjects were relatively low because of one-on-one training and counseling. Thus, we don’t know whether the combined contraceptive regimen can improve contraceptive effectiveness among a population who has a high rate of non-condom use and/or incorrect use of condoms. Therefore, further studies need to be conducted in different populations and with different methods.

## Conclusion

This study provides considerable evidence that male condom use remains a highly effective contraceptive method for a period of one year if consistently and correctly used. Providing training and counseling to subjects about condom use might be crucial to encouraging the consistent and correct use of condoms. Subjects need more information and guidance about how to correctly and consistently use condoms, which contributes to increasing the contraception efficacy of condoms.

### Ethical considerations

The study protocol and related instruments were reviewed and approved by the Institutional Review Boards (IRBs) of the World Health Organization (WHO) and the Shanghai Institute of Planned Parenthood Research (SIPPR). CONSORT guidelines were used in reporting the results of this research. Ethical issues (including plagiarism, informed consent, misconduct, data fabrication and/or falsification, double publication and/or submission, redundancy, etc.) have been completely observed by the authors. In addition, we provided guidance and assistance to the subjects who experienced unwanted pregnancy in our study groups.

## Competing interests

The authors declare that they have no competing interests.

## Authors’ contributions

RZ performed the statistical analysis, was involved in interpretation of the results, and drafted the manuscript. JQW and YYL conceived of the study and participated in the design of the study. YZ, HLJ, and YRL helped to do the statistical analysis and draft the manuscript. All authors read and approved the final manuscript.

## Pre-publication history

The pre-publication history for this paper can be accessed here:

http://www.biomedcentral.com/1471-2458/14/354/prepub

## References

[B1] Trussel J Kost K Contraceptive failure in the United States: critical review of the literature Stud Fam Plann 1987 18 5 237 283 10.2307/1966856 3318006

[B2] Jones EF Forrest JD Contraceptive failure in the United States: revised estimates from the 1982 national survey of family growth Fam Plann Perspect 1989 21 3 103 109 10.2307/2135659 2668019

[B3] Steiner M Kwok C Pregnancy risk among oral contraceptive pill, injectable contraceptive, and condom users in Uganda, Zimbabwe, and Thailand Obstet Gynecol 2007 5 11 1003 1009 17978111 10.1097/01.AOG.0000268804.98744.2b

[B4] Gallo MF Grimes DA Schulz KF Nonlatex vs. latex male condoms for contraception: a systematic review of randomized controlled trials Contraception 2003 68 319 326 10.1016/j.contraception.2003.08.015 14636934

[B5] Walsh TL Frezieres RG Peacock K Nelson AL Clark VA Embase BL Evaluation of the efficacy of a nonlatex condom: results from a randomized, controlled clinical trial Perspect Sex Reprod Health 2003 35 2 79 86 12729137

[B6] Richters J Donovan B Gerofi J Watson L Low condom breakage rates in commercial sex [**Letter**] Lancet 1988 2 1487 1488 2904595 10.1016/s0140-6736(88)90958-0

[B7] Walsh TL Ron G Peacock K Nelson AL Clark VA Bernstein L Wraxall BG Effectiveness of the male latex condom: combined results for three popular condom brands used as controls in randomized clinical trials Contraception 2004 70 407 413 10.1016/j.contraception.2004.05.008 15504381

[B8] Bankole A Singh S Hussain R Condom use for preventing STI/HIV and unintended pregnancy among young men in Sub-Saharan Africa Am J Men Health 2009 1 3 60 78 10.1177/1557988308322394 19477720

[B9] Crosby RA DiClemente RJ Wingood GM Salazar LF Rose E Levine D Brown L Lescano C Pugatch D Flanigan T Fernandez I Schlenger W Silver BJ Condom failure among adolescents: implications for STD prevention J Adolescent Health 2005 36 6 534 536 10.1016/j.jadohealth.2004.05.007 15901520

[B10] Paul KJ Garcia PJ Giesel AE Holmes KK Hitti JE Generation C: prevalence of and risk factors for Chlamydia trachomatis among adolescents and young women in Lima, Peru J Womens Health (Larchmt) 2009 18 9 1419 1424 10.1089/jwh.2008.1069 19698033 PMC2825717

[B11] Davis KR Weller SC The effectiveness of condoms in reducing heterosexual transmission of HIV Fam Plann Perspect 1999 31 272 279 10.2307/2991537 10614517

[B12] Hoefnagels C Hospers H Hosman C One measure, two motives. prediction of condom use and interaction between two prevention goals among heterosexual young adults: preventing pregnancy and/or sexually transmitted diseases Prev Sci 2006 7 369 376 10.1007/s11121-006-0048-z 16823634

[B13] Shanghai Municipal Health and Family Planning Commission Constituent ratio of contraceptive measures in Shanghai in 2008 2008 http://www.popinfo.gov.cn/dr/stat/ssh/20090611/0000000035000410011343697.html?openpath=spfp/stat/ssh

[B14] Shanghai Municipal Health and Family Planning Commission Constituent ratio of contraceptive measures in Shanghai in 2012 2012 http://www.popinfo.gov.cn/dr/stat/ssh/201352/000000003500041001134726694.html?openpath=spfp/stat/ssh

[B15] Polis CB Raymond EG Trussell J Advance provision of emergency contraception for pregnancy prevention Cochrane Database Syst Rev 2007 2 CD005497 17443596 10.1002/14651858.CD005497.pub2 PMC11270638

[B16] World Health Organization Improving Access to Quality Care in Family Planning Medical Eligibility Criteria for Contraceptive Use 2009 4 http://whqlibdoc.who.int/publications/2010/9789241563888_eng.pdf

[B17] Norris Turner A Ellertson C How safe is emergency contraception? Drug Saf 2002 25 10 695 706 10.2165/00002018-200225100-00002 12167065

[B18] McNeill E Gilmore C Finger W Lewis J Schellstede W The Latest Condom—Recent Advances Future Directions. FHI 1998

[B19] Murphy JS The Condom Industry in the United States 1990 Jefferson, NC: McFarland & Company, Inc.

[B20] Lytle CD Carney PG Vohra S Cyr WH Bockstahler LE Virus leakage through natural membrane condoms Sex Transm Dis 1990 17 58 62 10.1097/00007435-199004000-00002 2163114

[B21] Rosenberg MJ Waugh MS Latex condom breakage and slippage in a controlled clinical trial Contraception 1997 56 17 21 10.1016/S0010-7824(97)00069-3 9306027

[B22] Cook L Nanda K Taylor D Randomized crossover trial comparing the eZon plastic condom and a latex condom Contraception 2001 63 25 31 10.1016/S0010-7824(00)00193-1 11257245

[B23] Callahan M Mauck C Taylor D Frezieres R Walsh T Martens M Comparative evaluation of three Tactylonk condoms and a latex condom during vaginal intercourse: breakage and slippage Contraception 2000 61 205 215 10.1016/S0010-7824(00)00096-2 10827335

[B24] Richard AC Stephanie AS William LY Graham CA Condom-use errors and problems – a neglected aspect of studies assessing condom effectiveness Am J PrevMed 2003 24 4 367 370 10.1016/s0749-3797(03)00015-1 12726876

[B25] Flavel D Male perspective(s) on condom use: contest of STI/HIV prevention in the University of Ghana community J Public Health Epidemiol 2011 3 1 17 27

[B26] Xia G Yang X Risky sexual behavior among female entertainment workers in China: implications for HIV/STD prevention intervention AIDS educ prev: official publication of the International Society for AIDS Education 2005 17 2 143 156 10.1521/aeap.17.3.143.62904 15899752

[B27] Lindemann D Harbke C Are written instructions enough? Efficacy of male condom packaging leaflets among college students Health Educ J 2013 72 80

[B28] Mukenge-Tshibaka L Alary Nassirou Geraldo M Lowndes C Incorrect condom use and frequent breakage among female sex workers and their clients Int J STD AIDS 2005 16 345 347 15949062 10.1258/0956462053888916

[B29] Free C Roberts LG Abramsky T Fitzgerald M Wensley F A systematic review of randomised controlled trials of interventions promoting effective condom use J Epidemiol Community Health 2011 65 2 100 110 10.1136/jech.2008.085456 19822557 PMC3009845

[B30] Duerr A Gallo M Warner L Jamieson D Kulczycki A Macaluso M Assessing Male condom failure and incorrect use Sex Transm Dis 2011 38 7 580 586 10.1097/OLQ.0b013e3182096b62 21278626

